# The complete mitochondrial genome of the poisonous mushroom *Trichoderma cornu-damae* (Hypocreaceae)

**DOI:** 10.1080/23802359.2022.2135393

**Published:** 2022-11-04

**Authors:** Hwa-Yong Lee, Jong Won Jo, Young-Nam Kwak, Hyun Lee, Hojin Ryu, Jwakyung Sung, Yoon-Sup So, Chang-Sun Kim, Jong-Wook Chung

**Affiliations:** a Department of Forest Science, Chungbuk National University, Cheongju, Republic of Korea; b Forest Biodiversity Research Division, Korea National Arboretum, Pocheon, Republic of Korea; c Department of Biology, Chungbuk National University, Cheongju, Republic of Korea; d Department of Crop Science, Chungbuk National University, Cheongju, Republic of Korea; e Department of Industrial Plant Science and Technology, Chungbuk National University, Cheongju, Republic of Korea

**Keywords:** *Trichoderma cornu-damae*, mitogenome, poisonous mushroom

## Abstract

*Trichoderma cornu-damae* is a poisonous mushroom that contains trichothecene mycotoxins. The complete mitochondrial genome of this mushroom was determined using next-generation sequencing. This mitogenome is a circular molecule 94,608 bp in length with a GC content of 27.94% and contains 15 protein-coding genes, two rRNA genes (rnl and rns), and 25 tRNA genes. Phylogenetic analysis placed *T. cornu-damae* in the family Hypocreaceae group, which includes the genus *Trichoderma*. The mitogenome of *T. cornu*-*damae* will contribute to our understanding of the phylogeny, taxonomy, and population genetics of this mushroom.

*Trichoderma cornu-damae* (Pat.) Z.X. Zhu & W.Y. Zhuang 2014, a poisonous mushroom belonging to the Hypocreaceae family, is found in Korea, Japan, China, and Java, and the fruiting body is similar to that of a red pencil or deer horn (Gonmori et al. 2011; Zhu and Zhuang 2014; Kim et al. 2016). The major toxins of *T. cornu-damae* were identified as trichothecene macrolides, especially satratoxin H, and its 12′-acetate, 13′-acetate, and 12′,13′-diacetate (Saikawa et al. 2001). In 1999, five people drank alcoholic drinks prepared with approximately 1 g of this poisonous mushroom and died two days later despite intensive treatment (Saikawa et al. 2001). In Korea, one person who ate this mushroom developed nausea, vomiting, and fever within three hours, showed hemorrhagic and necrotic changes in the lungs, and died within 12 days due to multiorgan failure (Jang et al. 2013). This mushroom is difficult to distinguish from the young fruiting bodies of the edible mushrooms *Ganoderma lucidum* and *Cordyceps militaris*; therefore, poisoning cases have occurred in Korea and Japan (Kim et al. 2016; Choe et al. 2018). This is the first study to report the complete mitogenome sequence of the lethal poisonous mushroom *T. cornu-damae* and the first report to determine the phylogenetic position of this mushroom.

The strain of *T. cornu-damae* (voucher number: KA19-0412C) used in this study was collected from the Korea National Arboretum (Pocheon, South Korea; N37°45′11.84″, E127°09′54.85″). This sample was classified using the morphological method as per Zhu and Zhuang (2014), and through phylogenetic analysis using the ITS region of the DNA sequence. DNA for mitogenome analysis was extracted from mycelia isolated from this fruit body. Fruit body specimens and mycelia were deposited and maintained at the Korea National Arboretum (https://kna.forest.go.kr/, Dr. Chang-Sun Kim, changsun84@korea.kr). Total genomic DNA was extracted from mycelia cultured in potato dextrose agar media for 30 days in a dark environment using a GenEX Plant Kit (GeneAll, Seoul, South Korea), following the manufacturer’s instructions, and sequenced using an Illumina HiSeq platform. High-quality paired-end reads (>20 Phred) were obtained after trimming and assembling de novo using the CLC genome assembler (v. 4.21, CLC Inc., Aarhus, Denmark). From the initially assembled contigs, those with sequences derived from the mitochondrial genome were further processed to generate a single draft sequence, as previously reported by Lee et al. (2018). The draft sequence was manually corrected and gap filled by mapping a series of paired-end reads. The final complete mitogenome sequence was annotated using GeSeq (https://chlorobox.mpimp-golm.mpg.de/geseq-app.html) (Tillich et al. 2017) and manual curation was performed using the Artemis annotation tool (Rutherford et al. 2000) with NCBI BLASTN searches.

The complete mitochondrial genome sequence (GenBank accession no. MW525445) of *T. cornu-damae* is a circular molecule 94,608 bp in length with a GC content of 27.94%. This mitogenome contained 15 protein-coding genes, 25 tRNA genes, and two rRNA genes (*rnl* and *rns*). The 15 conserved protein-coding genes included seven subunits of NADH dehydrogenase (*nad1*, *nad2*, *nad3*, *nad4*, *nad4L*, *nad5*, and *nad6*), three subunits of cytochrome c oxidase (*cox1*, *cox2*, and *cox3*), three subunits of ATPase (*atp6*, *atp8*, and *atp9*), apocytochrome b (*cob*), and ribosomal protein S3 (*rps3*). The 25 tRNA genes covered all 20 standard amino acids and ranged in size from 70 bp to 86 bp.

For phylogenetic analysis, the DNA sequences of 11 coding genes (*atp6*, *atp8*, *atp9*, *cob*, *cox1*, *cox2*, *nad2*, *nad3*, *nad4*, *nad5*, and *nad6*) in the mitogenome of *T. cornu*-*damae* and 15 other fungal species were used. The general time reversible (GTR) model and maximum-likelihood method of MEGA 7 with 1000 bootstrap replicates were used (Kumar et al. 2016). Phylogenetic analysis placed *T. cornu-damae* into the family Hypocreaceae, which includes the genus *Trichoderma*, in comparison to other species (Figure 1). The mitogenome of *T. cornu-damae* reported in this study will contribute to the understanding of its phylogeny, taxonomy, and population genetics.

**Figure 1.**
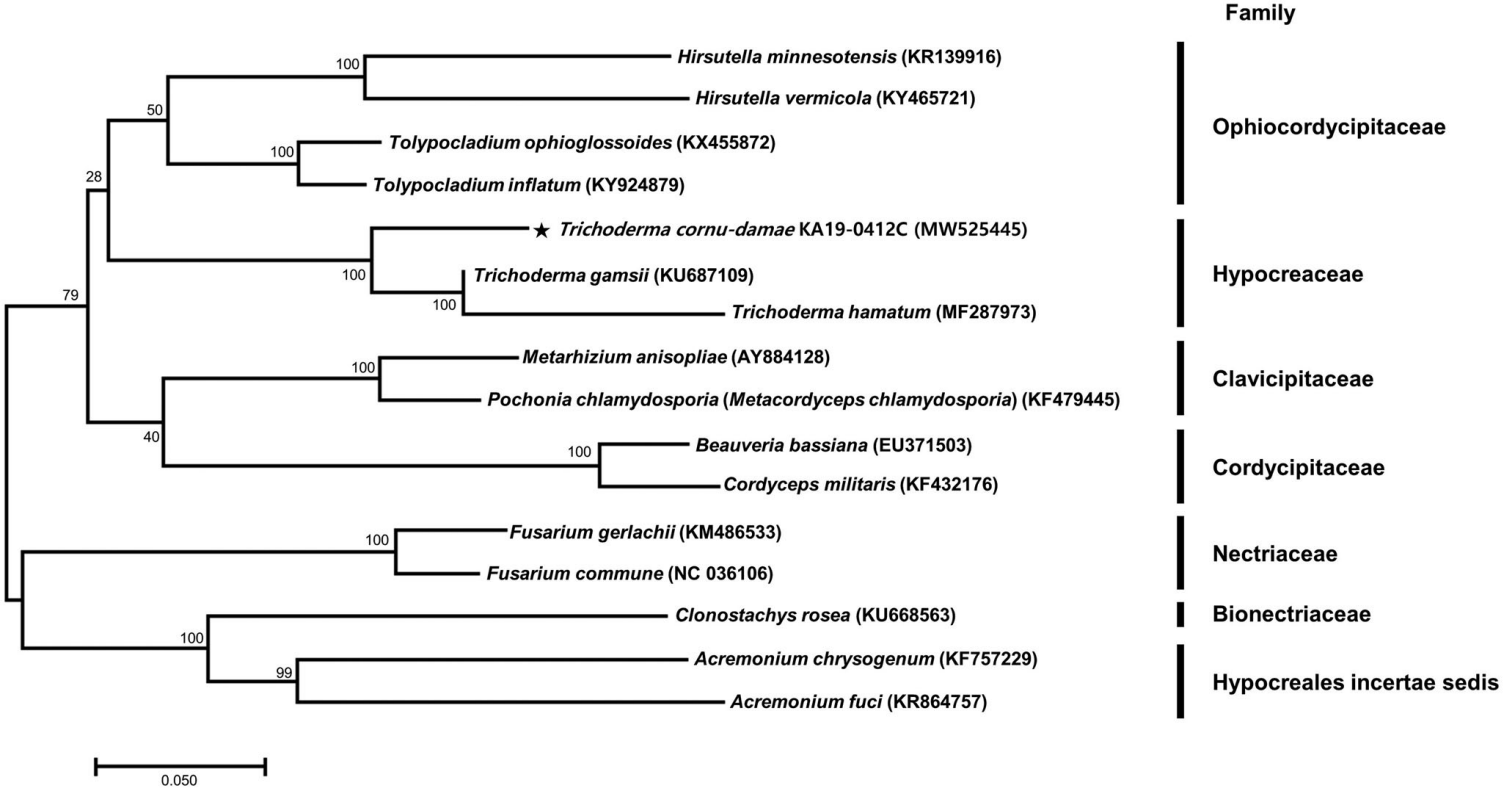
Maximum-likelihood phylogenetic tree based on DNA sequence of 11 coding genes (*atp6*, *atp8*, *atp9*, *cob*, *cox1*, *cox2*, *nad2*, *nad3*, *nad4*, *nad5*, and *nad6*) in the mitogenome of *Trichoderma cornu*-*damae* and other fungal species using MEGA 7 with maximum-likelihood method (Kumar et al. 2016).

## Data Availability

The data that support the findings of this study are openly available in GenBank of NCBI at https://www.ncbi.nlm.nih.gov/ under the accession no. MW525445. The associated BioProject, SRA, and Bio-Sample numbers are PRJNA739541, SRR18250171, and SAMN19791815, respectively.
